# Scoping of pharmacists’ health leadership training needs for effective antimicrobial stewardship in Africa

**DOI:** 10.1186/s40545-023-00543-2

**Published:** 2023-03-02

**Authors:** Ifunanya Ikhile, Gizem Gülpınar, Ayesha Iqbal, Nduta Kamere, Beth Ward, Manjula Halai, Amy Hai Yan Chan, Eric Muringu, Derick Munkombwe, Mashood Lawal, Winnie Nambatya, Yvonne Esseku, Felix Kaminyoghe, Shuwary Barlatt, Eva Muro, Chikondi Savieli, Diane Ashiru-Oredope, Victoria Rutter

**Affiliations:** 1 Commonwealth Pharmacists Association, London, E1W 1AW UK; 2 Department of Pharmacy Management, Faculty of Pharmacy, Gazi University, 06330 Ankara, Türkiye; 3 Division of Pharmacy Practice and Policy, School of Pharmacy, University of Nottingham, Nottingham, NG7 2RD UK; 4 Projects Department, Pharmaceutical Society of Kenya, P.O. Box 44290-00100, Nairobi, Kenya; 5 Pharmacy Department, University of Zambia, P.O. Box 50110, Lusaka, Zambia; 6 Pharmaceutical Society of Nigeria, P.O. Box 531, Lagos, Nigeria; 7 Pharmacy Department, College of Health Sciences, Makerere University, P.O. Box 7062, Kampala, Uganda; 8 Ghana College of Pharmacists, P.O. Box CT 10740, Accra, Ghana; 9 Pharmaceutical Society of Malawi, P.O. Box 2240, Lilongwe, Malawi; 10 Drug Information Services and Quality Assurance Unit, Directorate of Pharmaceutical Services, P.O. Box 232, Freetown, Sierra Leone; 11 Department of Pharmacology, Kilimanjaro Christian Medical University, Kilimanjaro, P.O. Box 2240, Moshi, Tanzania; 12 School of Medicine, Queens Medical Centre, University of Nottingham, Nottingham, NG7 2UH UK; 13 Office of Lifelong Learning and the Physician Learning Program, Faculty of Medicine and Dentistry, University of Alberta, AB T6G1C9 Edmonton, Canada

**Keywords:** Antimicrobial resistance, Antimicrobial stewardship, Commonwealth pharmacists Association, Health leadership, Pharmacists

## Abstract

**Background:**

Antimicrobial resistance (AMR) is a global public health concern currently mitigated by antimicrobial stewardship (AMS). Pharmacists are strategically placed to lead AMS actions that contribute to responsible use of antimicrobials; however, this is undermined by an acknowledged health leadership skills deficit. Learning from the UK’s Chief Pharmaceutical Officer's Global Health (ChPOGH) Fellowship programme, the Commonwealth Pharmacists Association (CPA) is focused to develop a health leadership training program for pharmacists in eight sub-Saharan African countries. This study thus explores need-based leadership training needs for pharmacists to provide effective AMS and inform the CPA’s development of a focused leadership training programme, the ‘Commonwealth Partnerships in AMS, Health Leadership Programme’ (CwPAMS/LP).

**Methods:**

A mixed methods approach was undertaken. Quantitative data were collected via a survey across 8 sub-Saharan African countries and descriptively analysed. Qualitative data were collected through 5 virtual focus group discussions, held between February and July 2021, involving stakeholder pharmacists from different sectors in the 8 countries and were analysed thematically. Data were triangulated to determine priority areas for the training programme.

**Results:**

The quantitative phase produced 484 survey responses. Focus groups had 40 participants from the 8 countries. Data analysis revealed a clear need for a health leadership programme, with 61% of respondents finding previous leadership training programmes highly beneficial or beneficial. A proportion of survey participants (37%) and the focus groups highlighted poor access to leadership training opportunities in their countries. Clinical pharmacy (34%) and health leadership (31%) were ranked as the two highest priority areas for further training of pharmacists. Within these priority areas, strategic thinking (65%), clinical knowledge (57%), coaching and mentoring (51%), and project management (58%) were selected as the most important.

**Conclusions:**

The study highlights the training needs of pharmacists and priority focus areas for health leadership to advance AMS within the African context. Context-specific identification of priority areas supports a needs-based approach to programme development, maximising African pharmacists’ contribution to AMS for improved and sustainable patient outcomes. This study recommends incorporating conflict management, behaviour change techniques, and advocacy, amongst others, as areas of focus to train pharmacist leaders to contribute to AMS effectively.

**Supplementary Information:**

The online version contains supplementary material available at 10.1186/s40545-023-00543-2.

## Background

Antimicrobial stewardship (AMS) involves a coherent set of actions that contribute to ensuring the responsible use of antimicrobials [1]. AMS combats Antimicrobial resistance (AMR), now recognised as a global public health concern, the ill effects of which are disproportionately experienced by low- and middle-income countries (LMICs) [2].

AMS can be undertaken by all health professionals at all levels. With pharmacists strategically placed, readily accessible, and often the first port of call for those seeking health services [3], they can make meaningful contributions towards optimising antimicrobial therapy and preventing AMR [4, 5]. Studies from various countries have widely reported pharmacists' role in AMS and their impact on societies [4, 5]; however, systemic, organisational and personal challenges, as well as an acknowledged health leadership skill deficit, have undermined this role [6, 7].

The importance of effective health leadership was especially felt during the COVID-19 pandemic [7]. It is integral to the role of pharmacists globally, essential for the delivery of high-quality healthcare services which improve organisational performance, staff satisfaction and retention [8] and, ultimately, enhance patient care outcomes [7]. However, pharmacists in LMICs often lack leadership training and education. Studies from sub-Saharan Africa report poor health systems leadership in AMS [5, 6], often leading to the inability of pharmacists to provide optimum patient-oriented services which can effectively tackle AMR. Enhancing health leadership skills will strengthen the pharmacy profession and positively influence the healthcare systems, societies, and patients with whom pharmacists engage.

Yet context-relevant health leadership training programmes for Africa are scarce. While literature is replete with research on generic leadership programmes, there is a paucity of approaches to designing and delivering healthcare training programmes within the African context [9].

The Department of Health and Social Care’s Fleming Fund is a UK aid programme supporting up to 25 countries across Africa and Asia to tackle AMR. The Fleming Fund supports the Commonwealth Partnerships for Antimicrobial Stewardship (CwPAMS) programme, managed by Commonwealth Pharmacists Association (CPA) and Tropical Health Education Trust (THET). CwPAMS is a health partnership programme with a clear aim to improve the rational use of antibiotics and achieve a subsequent reduction in morbidity and mortality associated with AMR in eight sub-Saharan African countries, which are Ghana, Kenya, Malawi, Nigeria, Sierra Leon, Tanzania, Uganda and Zambia. The CPA is developing a health leadership programme under the CwPAMS (CwPAMS/LP) to further support building capacity within the African pharmacist workforce, who are still being underutilised to address AMR [2].

The International Pharmaceutical Federation (FIP), World Health Organisation (WHO), and United Nations Educational, Scientific and Cultural Organisation (UNESCO) unanimously agree that the educational design and approach to developing the capability of pharmacists to deliver care in a variety of settings (e.g., community, hospital, research and development, academia), regardless of the stage of education (e.g., undergraduate, postgraduate, lifelong learning) must be quality driven and directed towards societal healthcare needs [10].

Hence, this study aimed to explore, scope and define context-specific, robust health leadership training for eight African countries to improve the capability of pharmacists to tackle AMR in Africa. Ensuring a needs-based approach by engaging stakeholders to identify important skills gaps in health leadership and to determine appropriate training delivery mechanisms and methods, thereby contextualising curricula to national priorities.

This was done to ensure fair representation, share decision-making ownership, and give due consideration to available resources, which will promote uptake and ensure that the implications of success are taken into account during implementation [11].

## Methods

### Country selection

Eight African countries, Ghana, Kenya, Malawi, Nigeria, Sierra Leon, Tanzania, Uganda and Zambia, were selected for data collection as they are part of the CwPAMS project under which the CPA is developing a health leadership programme (CwPAMS/LP).

### Study design

A participatory action research mixed methods approach was adopted in this study. Quantitative data were collected via a survey, and virtual focus group discussions were held with participants, who are the stakeholders of CPA, involved in AMS in the 8 African countries. The data were triangulated to obtain a 360-degree perspective on a fit-for-purpose, context-specific health leadership training programme.

## Quantitative study

### Sampling method

A random sampling method was adopted for this phase of the study and the minimum sample size was calculated as 379; on 0.05 significance level, *t* = 1.96, d (sensitivity) = 0.05, and *p* and *q* value, being 0.5 [12].

### Data collection

A questionnaire was designed to explore stakeholder perspectives on health leadership training needs [13]. The questionnaire was developed by three authors (II, VR, DA) of this study based on a literature review and expert opinion. The survey was piloted on a subset of the intended pharmacist population before the study (*n* = 30) to assess the interface, flow, importance, relevance, completion time, ease of completion, and comprehensibility (content, construct and face validity). Pilot feedback revealed that most participants found the questionnaire comprehensible and easy to follow. A few pilot participants suggested that clinical pharmacy was a significant training gap area that needed to be addressed; hence it was included in the final survey sent out to participants. The pilot results have not been included in this study.

The finalised questionnaire consisted of six sections (Additional file 1). The first and second sections relate to consent and basic participant demographics. The third section consisted of 4 questions exploring pharmacists' experiences and access to similar leadership courses and online learning platforms, if any. It evaluated the usefulness of health leadership courses and mentorship schemes using a 7-Likert scale from “highly beneficial” to “not beneficial”. Similarly, accessibility of online learning platforms was assessed using a 7-Likert scale starting with “very accessible” to “not accessible”. One question aimed to understand the preferred approach to accessing development opportunities. The fourth section explored the participants' expectations regarding the level of expertise they would develop after training, their priority areas for training, and developmental needs required to enable health leadership roles. One question focused on their perception of the relevance of the programme to their areas of current practice, such as AMS, clinical pharmacy, health leadership, service development/improvement and pharmaceutical public health. The fifth section explored the self-assessment of pharmacists’ competencies within the proposed training by asking them to rank their level of competence using a 9-Likert scale from “no competency” to “high competency”. The sixth section was designed to explore preferences for the delivery of a CwPAMS/LP, such as the expected duration of the programme and preferred training/learning delivery methods. The survey was designed to be completed within 15 min on average. No participant-identifying information was collected.

### Participant recruitment

The two phases of this study adopted stakeholder theory [14] to identify, invite and recruit participants across the 8 CwPAMS countries to ensure representativeness. Survey Monkey® (Momentive, California) was used to disseminate the survey to participants in all countries. CPA disseminated the survey through its in-country consultants, who were key liaison persons within each respective country regarding CPA’s AMS programmes. These in-country consultants disseminated the survey to local pharmacists using internal and external (social media) communication channels. The survey link remained open for 8 weeks from April to May 2021, and 3 weekly reminders were sent via CPA in-country consultants across their participant recruitment channels to improve participation. No inconvenience allowance was provided to the participants in this study.

### Data analysis

The survey responses were analysed using descriptive statistics via Survey Monkey® and Microsoft Excel®.

## Qualitative study

### Data collection

An interview guide was developed from the survey’s main themes for focus group discussions. However, content from the literature and expert consultation also enriched the contents of the topic guide (Additional file 2).

A purposeful critical case sampling method was used to ensure the diversification of participants and inclusion of stakeholders from mid-career to top-ranked high-influential policymakers across all 8 countries—perspectives required to guide the development of the CwPAMS/LP training programme. Participants were invited to participate in the study by the CPA in-country consultants. All participants in the focus group discussions had either completed or seen the survey to ensure a focused discussion.

All participants in the focus group discussions gave consent to be video recorded over Zoom® (Zoom Inc, USA). Five focus group discussions (approximately 50–90 min) were conducted with 5–12 pharmacists in each group from either one or two countries, at a date and time that was mutually convenient. The group numbers were limited to a maximum of 12 to encourage active participation [15]. Focus group discussions were conducted by one author (II) and moderated by others (MH, BW). No incentives were provided for participation in the study. The focus group data were collected until no new data was being obtained in subsequent focus groups.

### Data analysis

Focus group discussions were transcribed verbatim and thematically analysed [16] using the content of the survey as a guide. Transcripts were anonymised by assigning participants internal study codes as follows: “FG” (for focus group) and the sequence number of the focus group and their practice area (e.g., FG-1-Community practice). Transcribing, reading, and re-reading the transcripts aided familiarity with the data, which was content coded and categorised into sub-themes and themes and triangulated with survey data. As this was an exploratory and descriptive service improvement study, no theoretical framework was developed.

### Triangulation

All interviews and focus groups were triangulated, and the findings of each have been jointly reported in the results section. Sub-group analyses were planned for each country (quantitative), if the focus group discussions (qualitative) reflected significant variation in the data being obtained from each country.

### Data trustworthiness and reflexivity

Authors extensively trained in qualitative research methods were involved in topic guide development, data collection and data analysis. This combined expertise introduced trustworthiness and rigour in this study. Both survey and focus group data were anonymised and shared with stakeholders as a draft report to ensure the accuracy of inferred data as a technique of member checking. There were no discrepancies identified or changes suggested by the participants.

### Consent forms, ethics, and permissions

All participants completing the survey and attending the focus group discussions provided informed consent either as a part of the survey or a pre-filled consent form prior to the focus group discussions. All participation in the survey and focus groups was voluntary, and all participants consented to use the data collected during this study for internal service quality improvement as well as research purposes. The study was deemed exempt from ethical approval as this was a service improvement study for CPA.

## Results

### Demographics of people participating in the survey

A total of 484 people participated in the survey (Table 1 and Additional file 1) from all 8 countries. About half of the participants were from Kenya (*N* = 269) due to the effective recruitment strategy, linked to registration, employed by the in-country consultant. 66 participants were from Nigeria, 38 from Zambia, 36 from Tanzania, 14 each from Ghana and Malawi, 6 from Sierra Leone and 11 participants did not specify their country of origin. There was representation of pharmacists from different sectors, including academia, community practice, hospital, the Ministry of Health and National Pharmacists Associations (NPAs). There was almost an equal representation of males and females. The results showed that the majority of respondents were mid-career pharmacists with line management responsibilities. This represented the initial target population for the training programme. The demographics of survey respondents are presented in Table 1.

**Table 1:** Demographics of participants from all eight countries who responded to the survey

Variable Category	Group	Frequency (*N*)	Percentage (%)
Age	18–24	6	1.24
	25–34	205	42.36
	35–44	170	35.12
	45–54	54	11.16
	55–64	41	8.47
	65 +	8	1.65
Gender	Male	271	56
	Female	212	44
	Prefer not to say	1	0.21
Country of origin	Ghana	14	2.89
	Kenya	269	55.58
	Malawi	14	2.89
	Nigeria	66	13.64
	Sierra Leone	6	1.24
	Tanzania	36	7.44
	Uganda	30	6.20
	Zambia	38	7.85
	Not specified	11	2.27
Sector of predominant practice	Hospital	245	50.62
	Community	109	22.52
	Admin/Regulatory	43	8.88
	Academia	34	7.02
	Industry	13	2.69
	NGO	24	4.96
	Other	16	3.31
Number of practice years	< 1 year	12	2.48
	1–2 years	47	9.71
	3–5 years	133	27.48
	6–10 years	106	21.90
	11–20 years	114	23.55
	> 20 years	72	14.88
Workplace position	Staff	173	35.82
	Manager	181	37.47
	Director	67	13.87
	Chief Executive Officer	15	3.11
	Other	47	9.73
	Prefer not to say	1	0.21
Number of years of experience in management	Nil	46	9.50
	< 1 year	42	8.68
	1–2 years	102	21.07
	3–5 years	142	29.34
	6–10 years	72	14.88
	> 10 years	80	16.53

### Demographics of people participating in focus group discussions

A total of 40 representatives from each of the 8 countries participated in semi-structured focus group discussions (Additional file 2). There was a gender balance amongst these participants, with 22 male and 18 female participants, as shown in Table 2.

**Table 2:** Demographics of the focus group discussion participants

Variable Category	Group	Frequency (*N*)	Percentage (%)
Gender	Male	22	55.0
	Female	18	45.0
Country of origin	Ghana	4	10.0
	Kenya	6	15.0
	Malawi	4	10.0
	Nigeria	6	15.0
	Sierra Leone	5	12.5
	Tanzania	5	12.5
	Uganda	4	10.0
	Zambia	6	15.0
Area of practice	Chief pharmacists of hospitals	8	20.0
	Mid-career pharmacists in hospitals	6	15.0
	Mid-career pharmacists in community practice	4	10.0
	Academic pharmacists	9	22.5
	Representatives from the NPAs	2	5.0
	Representatives from health ministries	1	2.5
	CPA In-country consultants	8	20.0
	AMS focal persons	2	5.0

*NPA* National Pharmacist Association, *CPA* Commonwealth Pharmacy Association, *AMS* Antimicrobial stewardship

### Main findings of this study

Survey findings have been reported in the paragraphs and figures below. Quotes from qualitative data to provide context to the survey results are shown in Table 3.

**Table 3:** Main themes and related sub-themes derived from the qualitative data

Themes	Sub-themes	Quotes
Programme Relevance (An established need for the programme)	Opportunities for training Sectoral differences in opportunities for attendance in trainings	*“The main focus should be how to use health leadership to achieve results, change behaviours and so on” (FG1-Academia)* *“I have never been a part of anything formal leadership-wise…majority of the time it was just using what you know and figure your way out” (FG3-Hospital practice)* *“I work in the private sector, I am not employed by the public sector, we have little or no opportunities for leadership training here” (FG4-Community practice)*
Programme Content (A focus on health leadership content/module development)	Appropriateness of pre-selected modules	*“I agree that all these areas you have chosen are very relevant, pharmacists need to train themselves to a level where they cannot be ignored, especially in some countries where there is interprofessional rivalry where pharmacists are not considered as knowledgeable or where they are considered as a threat to medical doctors” (FG1-Health Ministry)* *“Pharmacists need to understand their role within the AMS space. This is their expectation for the training. To show them how pharmacists can contribute to AMS”(FG1-Hospital practice)*
Programme Delivery (A blended learning approach)	Issues with internet connectivity Blended approach to overcome time management with full-time jobs Need for engagement: Incentives Need for institutional buy-in	*“As a pharmacy educator, having live online sessions during the pandemic was not beneficial for every student because we have issues with internet connectivity, so if it is structured in a modular form where students can complete at their own time and pace and be examined after the module, I think that’s a better approach” (FG3-Academia)* *“If we can blend that would be the best, because there are certain aspects that cannot be adequately covered online, like when it comes to practice, it will be very difficult to transfer some of those skills” (FG2-Academia)* *“In our own context if we have to pay for the programme, it will be more of a problem. Its like having a scholarship, will you not engage if you get a scholarship? If people have to pay, I don’t think they will go in for it” (FG4-Academia)* *“In these parts, you may unravel things that we need to improve so you need to engage from management to the unit level for them to understand that it is for our own good we are not here to expose your weaknesses and it increases their participation in subsequent interventions” (FG3-AMS lead)*

### Programme relevance

Of the total, 83% of survey respondents found the proposed health leadership courses either highly beneficial or beneficial to their professional development. Health leadership and clinical pharmacy modules were ranked of highest relevance to pharmacists’ current practice, albeit marginally to the other modules (AMS, service development/improvement and pharmaceutical public health). This ranking was further validated by focus group participants, who indicated health leadership as a major gap area, as depicted in Table 3. A proportion of survey respondents (37%) had not attended in-country leadership training programmes, and 43% had not been part of formal mentorship schemes. Participants in focus group discussions attributed this primarily to a lack of access to national or other training opportunities (Table 3).

All focus group participants expressed a high demand for structured, high-quality health leadership training opportunities. It was reported that compared to pharmacists working in the community and the private sector, those employed in public institutions, especially in the hospital and academic sectors, have more access to organisational or institutional-level induction and training opportunities in managerial skills. Of the survey respondents, 16% reported previous ad-hoc access to professional development support through private companies, young pharmacists’ groups, NPAs, faith-based organisations, online research partnerships, professional networks, WhatsApp groups, colleagues, and non-governmental organisations (NGOs). Within the community pharmacy context, lack of access to training opportunities was highlighted as a significant barrier for mid-career pharmacists to engage in AMS.

The survey results also showed that training opportunities were mainly sought and accessed by the respondents themselves (67%) rather than through their employers (18%), the government (13%) or foreign organisations (8%), such as the CPA. Focus group discussions highlighted that pharmacists in LMICs would benefit from approaches to AMS knowledge and capacity building in accordance with standards practised in advanced countries like the United Kingdom. This could equip pharmacists with the necessary skills for delivering effective AMS services, which might help achieve desired service outcomes.

**Fig. 1:**
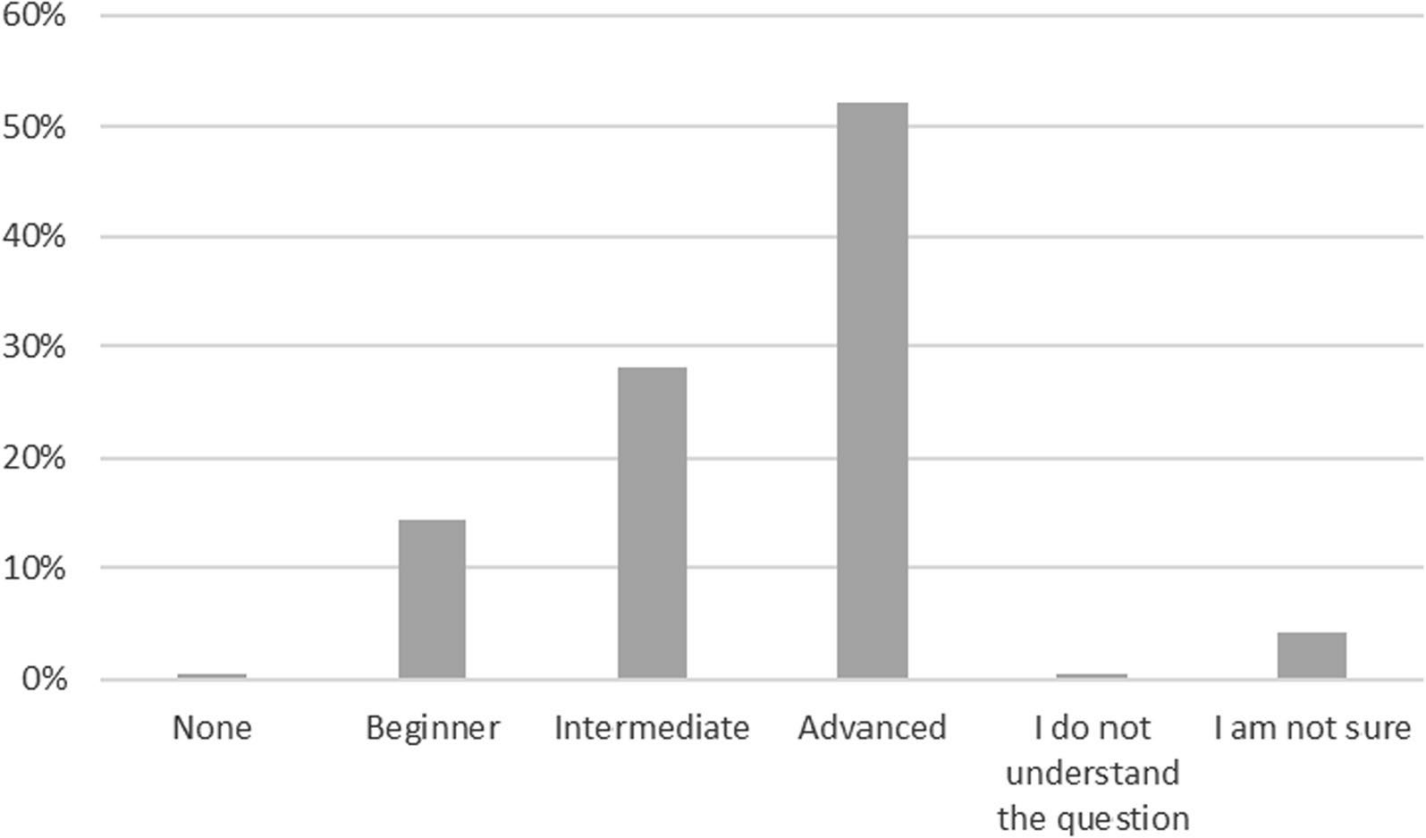
Level of expertise the pharmacists expect to achieve from the health leadership training

Both survey and focus group participants reported that upon completion of the health leadership training programme, they would expect to be practising at an advanced level, as the modules were perceived to provide the pharmacists with the required skills and competencies needed for an advanced health leader, as shown in Fig. 1.

### Training programme content

Overall, survey and focus group participants deemed the following modules appropriately reflected their training needs: health leadership, AMS, clinical pharmacy, service development/improvement and pharmaceutical public health. In addition, it was recommended to include research skills training in the programme, to support AMS leaders in contributing to evidence generation.

**Fig. 2:**
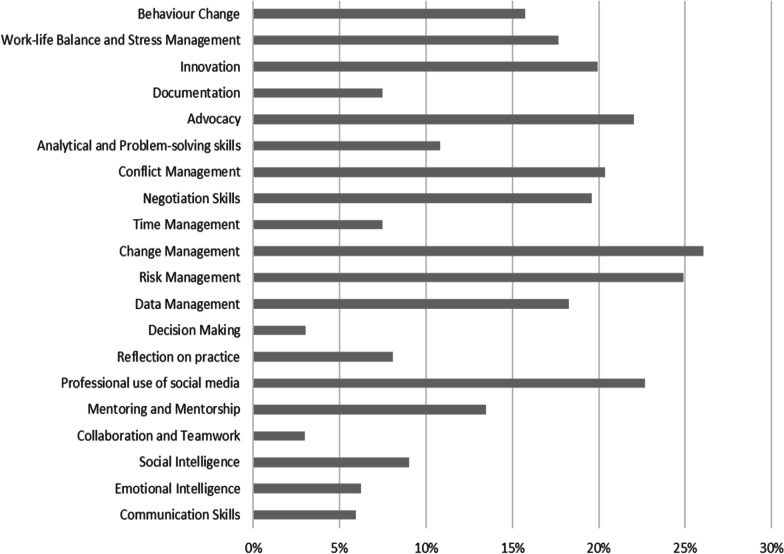
Priority training needs based on pharmacist–self-perceived lack of competence

Priority areas within these modules were determined based on areas of low competence in both the survey and discussions. Within the health leadership module, as shown in Fig. 2, change management (26%), risk management (25%), professional use of social media (23%), advocacy (22%), conflict management (20%), innovation (20%), data management (18%), work–life balance and stress management (18%), and behaviour change (16%) were recommended as areas, where pharmacists would benefit from further training. Participants in the focus groups also suggested that governance and audit training be prioritised.

Survey responses also showed that only 4% of respondents felt confident in their knowledge of the rational use of antimicrobials. Within the AMS module, suggested areas of focus included: how to develop an antibiogram (38%), epidemiology of AMR (36%), using AMS data for action (35%), diagnostic stewardship and surveillance (34%), a multidisciplinary approach to AMR (31%), and knowledge of national and global AMS policies (31%). Focus group participants also suggested that AMS training needs should be focused across hospital settings in their country (Table 3).

Within the pharmaceutical public health module, survey respondents felt they would benefit from knowledge and skills development in relation to mental health (40%), climate change (36%), epidemic preparedness (36%), as well as sustainability (30%) and health inequalities (28%).

Within the clinical pharmacy module, population health (31%), nutrition support (29%), research (26%), therapeutic drug monitoring (21%) and medical record management (20%) were identified as areas of low competence. Moreover, focus group participants suggested that training focused on these clinical pharmacy areas could help improve the quality of health services delivered by pharmacists as health leaders.

Within the service development/improvement module, project management (27%), the role of E-health (35%), programme evaluation (33%), policy development (33%) and clinical audit (27%) were areas of low competence.

### Programme delivery

The programme has been designed for online delivery as a pragmatic approach to running a programme across 8 countries, in particular as this was conducted during the COVID-19 pandemic’s travel restrictions. Respondents were familiar with online training platforms, having used them before and more frequently during the pandemic.

Many survey respondents (32%) believed online platforms were accessible. However, some concerns were raised during the focus group discussions, suggesting barriers such as poor internet service, patchy network coverage in several areas and the cost of data bundles required for training as shown in Table 3. This was validated during the study itself by focus group participants' difficulty in maintaining a connection during discussions. Other focus group participants, while acknowledging internet connectivity challenges, believed they were minor and could easily be overcome. The focus group participants did not make recommendations for any significant changes to the delivery of the training programme (Table 3).

Among the survey respondents, 42% recommended that the training programme duration should be 4–6 months, while 33% recommended 1–3 months. Some (17%) respondents suggested 10–12 months would be suitable. Focus group participants discussed the duration and concluded that up to 3 months might be too short to cover the intended modules, whereas 12 months may be too long and risked a high drop-out rate or disengagement of learners. A 6-month training period was agreed as most appropriate. The majority (35%) of survey respondents reported being able to commit 1–3 days per week to the programme, including at-work activities for the programme. This was the most selected response option. No survey respondents or focus group participants raised lack of time as a barrier to engaging with the programme, which may indicate it is perceived as being of high value.

**Fig. 3:**
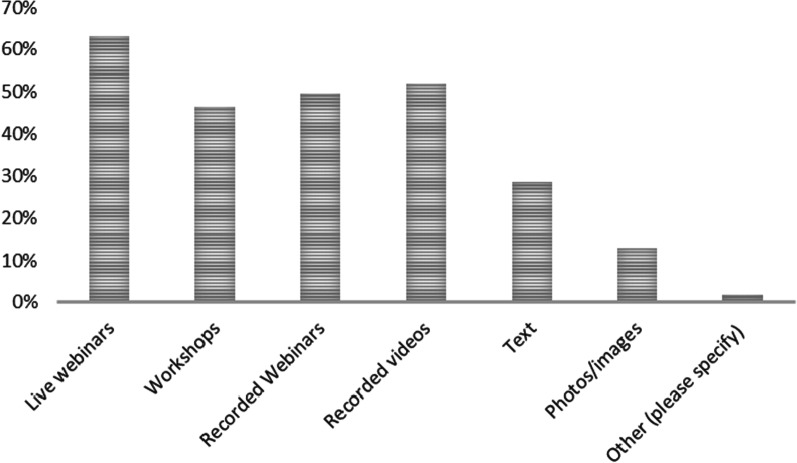
Preferred learning methods on an online platform

Regarding programme delivery, the majority of survey respondents (62%) preferred live webinars, followed by recorded webinars (50%), recorded videos (52%) and digital workshops (46%), as shown in Fig. 3. Focus group participants highlighted that time availability varies for individuals; therefore, live sessions should be limited in number and recorded sessions, and self-paced learning prioritised to maximise engagement. Therefore, a blended learning approach was suggested, incorporating self-paced online and face-to-face sessions (Table 3).

In relation to the end of programme assessment methodology, 84% of survey respondents preferred multiple-choice questions, and oral assessments being least preferred (18%).

The three preferred incentives for engagement and completion of the programme and assessment were certificates (74%), recognition from their NPA (64%), and networking and collaborative partnerships (64%). Gaining continuous professional development (CPD) points was also highly ranked, with 53% of respondents selecting this option. Interestingly, no respondents selected the option of completion resulting in membership of a ‘global leadership academy’. This could indicate a preference for social recognition over association or membership. Other incentives mentioned during focus group discussions included post-nominal titles to demonstrate expertise, induction into a network of experts, assurance of career progression, and NPA accreditation of the programme. Fee-free access to the training programme was also seen as a significant incentive (Table 3).

Participants recommended that engagement with institutional/organisation leadership teams by CPA would enhance local organisational buy-in and support for the programme.

## Discussion

This scoping study has been identified as the first of its kind. It explored areas of pharmacist competence as well as priority training needs to upskill the pharmacy workforce, building AMS health leadership capacity within the African pharmacy context. The study has informed the design of a contextualised training programme for pharmacists in eight sub-Saharan African countries, which aims to empower pharmacists through health leadership skills, to enhance AMS capacity. It is expected that identification of pharmacists’ relative needs and design of training accordingly, will enhance the impact of the intervention upon implementation [17].

This study did no sub-group analysis for different countries data, because the purpose of this study was to explore health leadership training needs which could train pharmacists across all 8 countries. This was also verified in focus group discussions, where participants did not comment on training material specific to any one country.

The majority of survey respondents and focus group participants reported no previous formal health leadership training, primarily due to lack of availability of accessible training in African countries. Where participants had attended any type of organisational or institutional leadership training or orientation, it was reported as highly beneficial, thereby establishing a desire for a structured, readily accessible programme. Focus group participants reported that poor access to training opportunities could be attributed to a lack of both government and pharmacy professional body focus on capacity building of pharmacists [18], resulting in a skills gap within the pharmacy workforce of these countries. This then impacts a pharmacist’s ability to consistently deliver the most effective health services and patient-focused interventions. Furthermore, this study highlights a need to explore the alignment of population needs with current pharmacy leadership education and training in the African continent. Pharmacy leadership development has been identified as a global need, as demonstrated by the inclusion of a leadership domain within FIP’s global advanced pharmacy framework. This framework, published following the commencement of this study, is designed to be adapted to local contexts, and the alignment of this study’s findings with the framework provides further validation of the study outcomes [19]. This study also emphasises the importance of health leadership to support the development of mid-career pharmacists in becoming local/team leaders, driving them to engage with and lead initiatives focused on improving public outcomes [19].

Both survey respondents and focus group participants had high expectations for the health leadership training programme, due to their previous positive learning experiences of CPA-organised CPD and other skills development training programmes. Health leadership, AMS, service development and pharmaceutical public health were identified and prioritised as core modules within this programme. Clinical pharmacy training modules were also identified as an area of training need for African pharmacists, similar to other countries [20–22]; however, developing generic clinical pharmacy training modules was deemed out of scope for this programme.

A qualitative study exploring the priorities and focus areas of NPAs across the Commonwealth showed a clear need and expectation of CPD programmes/training to be contextualised and provide pharmacists with relevant leadership training [23]; however, no studies from African countries which assessed the expectations of pharmacists on CPD training delivery were identified. This study shows that participants mostly self-identified training opportunities rather than relying on the government, employers, and professional associations such as CPA as a route to access. This may be due to the lack of awareness of pharmacists’ need to request external support for CPD. Participants in focus group interviews considered the role of CPA in hosting this programme as important, as CPA is believed to have the networks, expertise, and desired motivation to deliver pharmacists competency development in health leadership for AMS. Further research on the access or delivery of training needs for professional development to improve pharmacy practice across LMICs needs to be further explored.

A US study on healthcare leadership development and training suggests that more programmes with improved accessibility, are required to increase the number of healthcare leaders and improve the general quality of clinical practice, education, and research capacity [24]. Effective healthcare leadership and management capabilities have been described in the literature [25] and remain in congruence with those identified in this study, such as skills in change management, governance, policy development, and audit. This study’s findings further support recent research on successful leadership, which suggests that empathy and the ability to develop others through mentoring and coaching are critical skills for effective leaders [24].

A 2018 study explored community pharmacists’ attitudes towards leadership and managerial skills and reported that a lack of reward or financial incentive received after improving their skills [26] may impact a pharmacist’s desire to engage in competency development. This is in contrast to this study’s findings, where no such barriers or reservations were reported. All stakeholders agreed that there was value in dedicating time to leadership development as it enhanced their professional capability. This study reports a further benefit of health leadership development, as being the enhancement of collaborative practice and teamwork, which supports the optimisation of service delivery. Other studies suggest that a lack of leadership capabilities among health professionals is a major cause of interprofessional discord between interdisciplinary healthcare teams, which is commonly reported as a barrier for pharmacy services across LMICs [27]. With AMR being a global challenge, the need for multi-level interventions using the skills and capabilities of all healthcare team members would be ideal; however, with a lack of collaborative skills, pharmacists might be underutilised, reducing the efficacy of AMS initiatives.

Survey respondents and focus group participants identified that trained leaders could receive social recognition, which would support career advancement. Literature also reports that the completion of development programmes enhances recruitment prospects, provides greater career satisfaction [24] and improves individual and organisational performance [28]. Formal training programmes are conventionally accessed through employers, scholarships, foundations, and grants [26].

Leadership training programmes that adopt diverse learning formats, such as blended learning, have a high success rate and are important for future health leadership roles [25]. The lack of widespread internet access for pharmacists has been reported to influence and undermine the success of web-based programs and the use of online pharmacist learning portfolios [20]. Although this study identified a need for blended training delivery approaches, a study looking at perceived and actual learning found no difference between purely online versus blended learning approaches [29].

The study suggests that both early and late-career pharmacists would benefit from leadership training. Another study [30], advocates for pharmacist training in both early and late careers, developing highly skilled healthcare leaders throughout career maturation.

It is important to highlight that this study not only describes health leadership skills development for AMS but highlights its advantages to achieving a generic skill set required for effective service delivery by pharmacists. It emphasises that health leadership skills should be adopted as “baseline” or “core” skills, which can then be applied in different clinical contexts. This flexible approach results in the programme being highly adaptable across several medication-related or disease-related pharmacy services for both hospital and community pharmacy practice, thereby supporting improved medication safety and optimised care delivery by pharmacists.

A key strength of this health leadership programme is its needs-based design. Contextualising the programme to the African pharmacy context increases the likelihood of positive and sustained impact.

The ultimate intent is to develop a bespoke programme for developing pharmacist leaders within clinical practice, to create a cadre of leaders who can contribute towards the sustainable improvement of the health system in their respective countries using acquired skills.

## Conclusion

The findings of this study informed the design and development of a tailored health leadership training programme contextualised to pharmacist training requirements in eight African countries. Core areas of focus for the programme include conflict management, behaviour change, advocacy, and building pharmacists’ capability to effectively contribute to AMR national and global policies. The training programme has been designed to support hospital and community pharmacists to improve their health leadership roles in AMS, with a view to successfully addressing AMR challenges in African countries.

## Supplementary Information

**Additional file 1. **Survey questionnaire.

**Additional file 2. **Focus group interview guide.

## Data Availability

All the data and materials used during the study are available on reasonable request to the corresponding author, CS.

## Abbreviations

AMR: Antimicrobial resistance
AMS: Antimicrobial stewardship
CPA: Commonwealth Pharmacists Association
CwPAMS: Commonwealth Partnerships in Antimicrobial Stewardship
CwPAMS/LP: Commonwealth Partnerships in AMS Health Leadership Programme
ChPOGH: Chief Pharmaceutical Officer's Global Health
CPD: Continuing professional development
LMICs: Low- and medium-income countries
FIP: International Pharmaceutical Federation
WHO: World Health Organisation
UNESCO: United Nations Educational, Scientific and Cultural Organisation
NPA: National Pharmacists Association
