# Supplementary material for: Scoping of pharmacists’ health leadership training needs for effective antimicrobial stewardship in Africa
Source: J Pharm Policy Pract. 2023 Mar 2;16:33. doi: 10.1186/s40545-023-00543-2 (PMC9979108; doi:10.1186/s40545-023-00543-2)
Supplement: Supplementary file 1 — Additional file 1. Survey questionnaire. [file 40545_2023_543_MOESM1_ESM.docx]

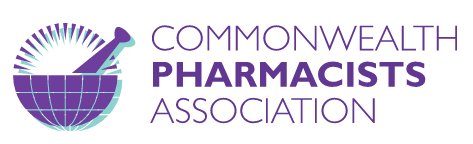


**Participant Information**

You are being invited to participate in this survey. Before you complete the survey, it is important for you to understand why this data is being collected and what your participation will involve. Completing this survey will be taken as consent to participate and for data provided to be used for programme development. No ethics approval is required for this survey as data collected is not considered to be sensitive or confidential and no vulnerable groups are involved. Please read the following information carefully, and kindly contact the email provided if anything is unclear, or if you would like more information. Thank you.

**What is this survey about?**

The Commonwealth Pharmacists Association (CPA) in keeping with its goal of capacity development for the pharmacy workforce and health system strengthening for member countries is currently planning a needs-based, fit-for-purpose health leadership training programme with funding from the UK Department of Health and Social Care’s Fleming Fund.

We understand that such a needs-based approach requires ongoing consultation and cooperative partnerships between all stakeholders within countries and institutions, so there can be shared ownership for decision making, implementation and outcomes. Hence this survey seeks to explore all stakeholders’ views on training needs.

The health leadership programme in development specifically focuses on capacity building of mid-career pharmacists within antimicrobial stewardship (AMS) and pharmaceutical public health while incorporating service development, quality improvement and behavioural change, as well to ensure sustainability of progress made.

**Why have you been invited?**

You have been invited because you are a practising pharmacist in your country and considered to be a stakeholder. Several other pharmacists across 8 African countries have also been invited to take part in this survey.

You will not be paid to participate in this survey but the information we get will help us understand how best to develop the training programme in such a way that participants are able to undertake leadership roles and apply knowledge and skills gained to generate creative solutions to real-life AMR issues within their institutions.

**Data Management**

We will follow ethical and legal practice, and all information about you will be handled in confidence. Results may be published but no individual comments or assignment of comments will be made. All responses to the questions in the questionnaire will also be anonymised.

Thank you for your participation.

**Questionnaire Section A (Demographics)**

1. **Gender**

- Male
- Female
- Prefer not to say

1. **Age**

- 18-24
- 25-34
- 35-44
- 45-54
- 55-64
- 65 and older

1. **Nationality**

- Ghana
- Kenya
- Malawi
- Nigeria
- Sierra Leone
- Tanzania
- Uganda
- Zambia
- Other (Please specify) ____________________

1. **Country of work**

- Ghana
- Kenya
- Malawi
- Nigeria
- Sierra Leone
- Tanzania
- Uganda
- Zambia
- Other (Please specify_____________________

1. **How long have you worked as a pharmacist?**

- <1 year
- 1-2 years
- 3-5 years
- 6-10 years
- 11-19 years
- >20 years

1. **Highest degree completed**

- Bachelor’s degree
- Master’s degree
- Doctor of Pharmacy (PharmD)
- Doctor of Philosophy (PhD)
- Other (Please specify) ________________________

1. **Sector of predominant practice**

- Hospital
- Community practice
- Admin/Regulatory
- Academia
- Industry
- NGO
- Other (Please specify) __________________________

1. **Position in workplace**

- Staff
- Manager
- Director
- CEO
- Other (Please specify) ___________________________

1. **How long have you had management responsibilities?**

- I do not manage anyone
- <1 year
- 1-2 years
- 3-5 years
- 6-10 years
- >10 years

**Questionnaire Section B (Professional development opportunities)**

**This section seeks to explore pharmacists’ access to similar training courses.**

**Usefulness of health leadership courses**

1. How beneficial have previous in-country health leadership* courses been to your professional development?

*Health leadership courses refer to any professional development course focused on building your capacity as a leader within healthcare.

- Highly beneficial
- Beneficial
- Moderately beneficial
- Somewhat beneficial
- Not beneficial
- I do not understand question
- I have not previously attended in-country health leadership courses

If applicable, please name the health leadership course and provider________________

**Usefulness of mentorship schemes**

1. How beneficial have previous in-country mentorship* programmes been to your professional development?

*Health mentorship schemes refer to arrangements where you have been assigned to an experienced person to guide your career or personal progress.

- Highly beneficial
- Beneficial
- Moderately beneficial
- Somewhat beneficial
- Not beneficial
- I do not understand question
- I have not been part of any in-country mentorship programmes

If applicable, please name the mentorship scheme and provider___________________

**Accessibility of online learning platforms**

1. How accessible are high quality online learning platforms for leadership development to you?

*Digital learning platforms refer to online environments enabled by electronic technology used for the purpose of training learning or development

- Very accessible
- Accessible
- Moderately accessible
- Somewhat accessible
- Not accessible
- I do not understand question
- I am not sure

If applicable, please name the online learning platform_____________________________

1. How did you gain access to all the development opportunities described above? **(multiresponse)**

- Self
- Employer
- Government
- Foreign Aid Organizations
- Other (Please specify) __________________________________

**Questionnaire Section C (Programme Relevance)**

**This section seeks to establish how suitable the current plan for the health leadership programme is.**

**Expectation**

1. What level of expertise do you expect from this training?

*At what level do you think you would be practising after training as a result of the knowledge and skills acquired

- None
- Beginner
- Intermediate
- Advanced
- Do not understand question
- I am not sure

1. What top 5 topics reflect your most important development and training needs? **(ranking)**

*Details of any area of health leadership that you feel you need training on.

- Presentation skills
- Communication skills
- Coaching and Mentoring others
- Change Management
- IT systems and new tech
- Project Management
- Personal Effectiveness
- Teamwork
- Management fundamentals
- Clinical Knowledge
- Time Management
- Assertiveness
- Impact
- Strategic Thinking

Other important training needs (please specify) _____________________________

**Module relevance**

1. Can you rank these modules in order of relevance to your practice starting with the most relevant?

- Health Leadership
- AMS
- Clinical pharmacy
- Service development/improvement
- Pharmaceutical public health

**Pharmacists’ Competence**

**Think about your current or previous job roles while answering the questions below.**

**Kindly choose responses to the statements below from the provided options (Strongly Agree, Agree, Neutral, Disagree, Strongly Disagree) to describe the extent to which they apply to you.**

I have acquired sufficient knowledge and skills to handle my current scope of work within the following areas.

|  | **Areas** | **Strongly Agree** | **Agree** | **Neutral** | **Disagree** | **Strongly Disagree** | **I am not sure** | **Do not understand question** | **Not applicable** |
| --- | --- | --- | --- | --- | --- | --- | --- | --- | --- |
|  | AMS/AMR |  |  |  |  |  |  |  |  |
|  | Health Leadership |  |  |  |  |  |  |  |  |
|  | Service Development |  |  |  |  |  |  |  |  |
|  | Pharmaceutical Public Health |  |  |  |  |  |  |  |  |

**Kindly choose responses to the topics below from the provided options (No competence, low competence, moderate competence, high competence and not applicable to job) to describe your level of competence within the domains listed.**

**Health Leadership competence**

What would you say your level of competence is within the following health leadership skills domains?

|  | **Domains** | **No Competence** | **Low Competence** | **Moderate Competence** | **High Competence** | **I am not sure** | **Not applicable to job** |
| --- | --- | --- | --- | --- | --- | --- | --- |
|  | Communication Skills |  |  |  |  |  |  |
|  | Emotional Intelligence |  |  |  |  |  |  |
|  | Social Intelligence |  |  |  |  |  |  |
|  | Collaboration and Teamwork |  |  |  |  |  |  |
|  | Mentoring and mentorship |  |  |  |  |  |  |
|  | Professional use of social media |  |  |  |  |  |  |
|  | Reflection on practice |  |  |  |  |  |  |
|  | Decision Making |  |  |  |  |  |  |
|  | Data Management |  |  |  |  |  |  |
|  | Risk Management |  |  |  |  |  |  |
|  | Change Management |  |  |  |  |  |  |
|  | Time Management |  |  |  |  |  |  |
|  | Negotiation skills |  |  |  |  |  |  |
|  | Conflict Management |  |  |  |  |  |  |
|  | Analytical and problem-solving skills |  |  |  |  |  |  |
|  | Advocacy |  |  |  |  |  |  |
|  | Documentation |  |  |  |  |  |  |
|  | Innovation |  |  |  |  |  |  |
|  | Work-life balance and stress management |  |  |  |  |  |  |
|  | Behaviour Change |  |  |  |  |  |  |

**AMS competence**

What would you say your level of competence is within the following AMS/AMR knowledge domains?

|  | **Domains** | **No Competence** | **Low Competence** | **Moderate Competence** | **High Competence** | **I am not sure** | **Not applicable to job** |
| --- | --- | --- | --- | --- | --- | --- | --- |
|  | Infection prevention and control |  |  |  |  |  |  |
|  | Rational use of Antimicrobials |  |  |  |  |  |  |
|  | National and International AMS policies |  |  |  |  |  |  |
|  | Epidemiology of AMR |  |  |  |  |  |  |
|  | Antimicrobial interactions and ADRs |  |  |  |  |  |  |
|  | Therapeutic Drug Monitoring |  |  |  |  |  |  |
|  | Diagnostic stewardship and surveillance |  |  |  |  |  |  |
|  | AWaRe |  |  |  |  |  |  |
|  | Developing AMS interventions |  |  |  |  |  |  |
|  | Using AMS data for action |  |  |  |  |  |  |
|  | Developing an antibiogram |  |  |  |  |  |  |
|  | Multidisciplinary approach to AMS |  |  |  |  |  |  |

**Pharmaceutical public health competence**

What would you say your level of competence is within the following Pharmaceutical Public Health domains?

|  | **Domains** | **No Competence** | **Low Competence** | **Moderate Competence** | **High Competence** | **I am not sure** | **Not applicable to job** |
| --- | --- | --- | --- | --- | --- | --- | --- |
|  | Health inequalities |  |  |  |  |  |  |
|  | Supply chain |  |  |  |  |  |  |
|  | Health Systems strengthening |  |  |  |  |  |  |
|  | Non-communicable diseases (NCDs)- cancer, diabetes |  |  |  |  |  |  |
|  | Infectious diseases- COVID, HIV, Malaria, TB |  |  |  |  |  |  |
|  | Access to medicines |  |  |  |  |  |  |
|  | Climate change |  |  |  |  |  |  |
|  | Mental health |  |  |  |  |  |  |
|  | Interprofessional collaboration |  |  |  |  |  |  |
|  | Epidemic preparedness |  |  |  |  |  |  |
|  | Sustainability |  |  |  |  |  |  |

**Clinical pharmacy competence**

What would you say your level of competence is within the following clinical pharmacy domains?

|  | **Domains** | **No Competence** | **Low Competence** | **Moderate Competence** | **High Competence** | **I am not sure** | **Not applicable to job** |
| --- | --- | --- | --- | --- | --- | --- | --- |
|  | Direct patient care |  |  |  |  |  |  |
|  | Pharmacotherapy |  |  |  |  |  |  |
|  | Population health |  |  |  |  |  |  |
|  | Professionalism |  |  |  |  |  |  |
|  | CPD (Continuing Professional Development) |  |  |  |  |  |  |
|  | Medical record management |  |  |  |  |  |  |
|  | Pathophysiology |  |  |  |  |  |  |
|  | Patient education |  |  |  |  |  |  |
|  | Medicines Management (Storage, dispensing etc) |  |  |  |  |  |  |
|  | Therapeutic drug monitoring |  |  |  |  |  |  |
|  | Adverse drug reaction reporting |  |  |  |  |  |  |
|  | Clinical Research |  |  |  |  |  |  |
|  | Patient counselling |  |  |  |  |  |  |

**Service development competence**

What would you say your level of competence is within the following Service development domains?

|  | **Domains** | **No Competence** | **Low Competence** | **Moderate Competence** | **High Competence** | **I am not sure** | **Not applicable to job** |
| --- | --- | --- | --- | --- | --- | --- | --- |
|  | Project Management |  |  |  |  |  |  |
|  | Programme Evaluation |  |  |  |  |  |  |
|  | Role of E-health |  |  |  |  |  |  |
|  | Resource management |  |  |  |  |  |  |
|  | Budgeting and cost effectiveness |  |  |  |  |  |  |
|  | Policy Development |  |  |  |  |  |  |
|  | Clinical audit |  |  |  |  |  |  |
|  | Research methods |  |  |  |  |  |  |
|  | Quality Improvement |  |  |  |  |  |  |

**Topics**

1. Are there other training topics you would like to see on the course?

Please specify_______________________________________________

**Duration**

1. A suitable duration for this programme would be

- 1-3 months
- 4-6 months
- 6-9 months
- 9-12 months
- More than a year

1. How much time do you think you can commit per week to this programme?

- 4-7 day
- 1-3 days
- 12-23 hours
- 6-11 hours
- 3-5 hours
- <3 hours
- No time

**Delivery**

1. Your preferred learning methods on an online platform include **(multiresponse)**

- Group discussions
- Workshops
- Webinars
- Recorded videos
- Text
- Other (please specify) _____________________________

**Assessment**

1. Your preferred post-programme assessment methods include **(multirepsonse)**

- MCQs
- Essay
- Viva/Oral assessment
- Group presentation
- Other (please specify) _____________________________

**Incentives**

1. What would incentivize you to complete this programme

- CPD training points
- Certificate
- Recognition/accreditation from your national pharmacy association
- Membership of a global leadership academy
- Networks and collaborative partnerships
- Other (please specify) _____________________________

**Survey dissemination**

1. How did you find out about this survey?

- Commonwealth Pharmacists Association
- National Pharmacy Association
- Employer
- Social Media
- Colleague/Fellow Pharmacist
- Other (Please specify) _________________

**Comments for general improvement**

1. Do you have any suggestions or comments that will help us make the programme better?

___________________________________________________________________________

**Contact information-**
