# Supplementary material for: Scoping of pharmacists’ health leadership training needs for effective antimicrobial stewardship in Africa
Source: J Pharm Policy Pract. 2023 Mar 2;16:33. doi: 10.1186/s40545-023-00543-2 (PMC9979108; doi:10.1186/s40545-023-00543-2)
Supplement: Supplementary file 2 — Additional file 2. Focus group interview guide. [file 40545_2023_543_MOESM2_ESM.docx]

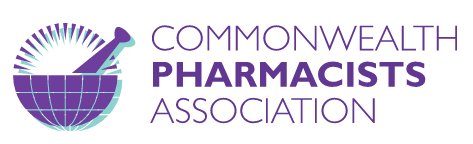


# 1.1 Participant Information

**Study Title:**

Stakeholders’ Perspectives on the Commonwealth Partnerships for Anti-microbial Stewardship and Health Leadership Programme (CwPAMS LP).

**Information Sheet for Focus Group Participants:**

You are being invited to be involved in this study. Before you decide whether you want to take part, it is important for you to understand why the focus group is being conducted and what your participation will involve.

Please take time to read the following information carefully and discuss it with other members of staff from your organization if you wish. Please contact me if anything is unclear or if you would like more information. Take time to decide whether or not you wish to take part. Thank you for reading this.

**Purpose of this Study:**

The Commonwealth Pharmacists Association (CPA) in keeping with its goal of capacity development for the pharmacy workforce and health system strengthening for member countries is currently planning a needs-based, fit-for-purpose health leadership training programme within 8 African countries with funding from the UK Department of Health and Social Care’s Fleming Fund.

We understand that such a needs-based approach requires ongoing consultation and cooperative partnerships between all stakeholders within countries and institutions, so there can be shared ownership for decision making, implementation and outcomes. Hence the focus groups seek to explore stakeholders’ views on the programme.

The health leadership programme in development specifically focuses on capacity building of mid-career pharmacists within antimicrobial stewardship (AMS) and pharmaceutical public health while incorporating service development, quality improvement and behavioural change, as well, to ensure sustainability of progress made.

**Study Aims:**

- To actively engage with key stakeholders within 8 countries on plans for the CwPAMS LP.
- To explore access to leadership training opportunities for pharmacists in these 8 countries.
- To determine stakeholders’ perspectives on pertinent training needs within AMS and health leadership domains.
- To identify potential facilitators and barriers to this programme, and to what extent they can influence its success.

This is being done with an understanding that collaboration with stakeholders is key to the success of the programme. Focus group interviews are being conducted concurrently with a survey exploring similar themes. It is hoped that the study will lead to a fit-for-purpose health leadership training programme by ensuring relevance to practice within the context of selected countries.

**Why you have been invited:**

You have been invited because you are a practising pharmacist in your country and considered to be a key informant. Several other pharmacists representing various practice sectors across 8 African countries have also been invited to participate in these focus groups.

You will not be paid for your participation, but the information we get will help us understand how best to develop the training programme in such a way that participants are able to undertake leadership roles and apply knowledge and skills gained to generate creative solutions to real-life AMR issues within their institutions.

**The Focus Group Interview:**

This focus group would be conducted online (on zoom). You should have received an invitation link (please let me know if you have not). It is one of four preliminary focus group meetings of locally practising stakeholders across the 8 countries. Countries have been paired in each focus group to generate richer data from different perspectives and provide better insight into the topic. The duration should be about 60-90 minutes. The sessions would be video recorded and later transcribed into text form.

The information we get from this study will help to better understand AMS and Health Leadership in the context of your country, and knowledge from themes will help to make evidence-based decisions on key domains for a needs-based, fit-for-purpose health leadership programme.

**Anonymity/Participation:**

We will follow ethical and legal practice and all information about you will be handled in confidence. If you participate in the focus group, some parts of the data collected for the study will be looked at by authorised persons from the Commonwealth Pharmacists Association during analysis. All will have a duty of confidentiality to you as a research participant and we will do our best to meet this duty. Your participation is voluntary, and you may withdraw from the research project at any stage, without having to give any reason, and withdrawing will not penalize or disadvantage you in any way.

**Data Management:**

All information which is collected about you during the course of the research will be kept **strictly confidential**, stored in a secure and locked office, and on a password protected database. Any information about you which leaves the organisation will have your name and email address removed (anonymised) and a unique code will be used so that you cannot be recognised from it. Your personal data (email address, telephone number) will be kept for twelve months after the end of the study so that we are able to contact you about the findings of the study *and possible follow-up studies* (unless you advise us that you do not wish to be contacted). All other data (research data) will be kept securely for 7 years. After this time, your data will be disposed of securely. During this time, precautions will be taken by all those involved to maintain your confidentiality. Only members of the research team will have access to your personal data.

As part of the presentation of results, your own words may be used in text form. This will be anonymised, so that you cannot be identified from what you said. Although what you say in the focus group interview is confidential, in the highly unlikely event that you disclose anything to us which we feel puts you or anyone else at any risk, we may feel it necessary to report this to the appropriate authority.

**Data Analysis and Dissemination**:

The end of this phase of the study will be the last focus group with the last set of participants. After which data analysis would be done by the research team in CPA. Interview transcripts will be sent to you upon request. A brief summary of the findings can be made available by the researcher upon request. It is also possible that the results will be presented at academic conferences and journals. The study sponsor and funder will be acknowledged in all publications.

# 1.2 Participant Consent Form

- I have read the Participant Information Sheet and the nature and purpose of the project has been explained to me. I understand and agree to take part.
- I understand the purpose of the study and my involvement in it.
- I understand my participation is voluntary and that I may withdraw from the project at any stage and that this will not affect my status now or in the future.
- I understand that while information gained during the study may be published, any information I provide is confidential (with one exception – see below), and that no information that could lead to the identification of any individual will be disclosed in any reports on the project, or to any other party. No identifiable personal data will be published.
- I agree that extracts from the focus group may be anonymously quoted in any report or publication arising from the study.
- I understand that though what I say in the focus group interview is confidential, the researcher may be required to report to the authorities, in the highly unlikely event that I disclose anything which puts me or anyone else at any risk.
- I understand that I will be recorded during the focus group interview.
- I understand that data will be stored in a secure and locked office, and on a password protected database. All research data will be kept securely for 7 years. After this time data will be disposed of securely. During this time precautions will be taken by all involved to maintain confidentiality.
- I understand that the information provided can be used in other similar projects within the CPA, but that my name and contact information will be removed before it is used.
- I understand that I may contact the researcher if I require further information about the research, and that I may contact the Commonwealth Pharmacists Association, if I wish to make a complaint relating to my involvement in the research.
- I agree to take part in the above research project.

**Signed** ………………………………………………………………………… (Focus Group Participant)

**Print name** …………………………………………………………………

**Date** …………………………………

This project is being organised by the Commonwealth Pharmacists Association and is being funded by the UK Department of Health and Social Care’s Fleming Fund. If you are concerned with any aspect of this study, you can contact the researcher, if you are still unsatisfied, please feel free to contact the Commonwealth Pharmacists Association using the details below for further advice and information:

**Researcher Contact:**

Dr Ifunanya Ikhile

Education Advisor

Commonwealth Pharmacists Association

ifunanya.ikhile@commonwealthpharmacy.org

**Chief Investigator Contact for further information:**

Victoria Rutter

Executive Director

Commonwealth Pharmacists Association

Victoria.rutter@commonwealthpharmacy.org

# 1.3 Focus Group Schedule

**Opening Statement:**

Good morning/afternoon, and welcome to our focus group session. Thank you for taking time out of your busy schedule to discuss the CwPAMS Leadership Programme.

My name is Ifunanya Ikhile, Education advisor at CPA, and we’ll just have a quick round of introductions.

The purpose of the focus group is to get some information from key stakeholders on what a fit-for-purpose AMS and Health leadership programme entails in the context of your specific country. We want to know what your experiences of previous leadership training programmes have been, what your AMS and health leadership training needs are, how you would like the programme to be run and what you foresee to be likely barriers and facilitators to the success of the programme. This information will aid the design of a fit-for-purpose AMS and health leadership programme to be run in 8 African countries (Ghana, Malawi, Zambia, Tanzania, Kenya, Nigeria, Uganda, and Sierra Leone).

This focus group is made of stakeholders from X and Y countries, and we are having similar discussions across the 8 countries. You were invited because you are a pharmacy stakeholder and are perceived to be a key informant.

This session will go on for between 60 and 90 minutes, and we would be grateful for enthusiastic participation. There are no wrong answers but differing points of view. So, please feel free to share your point of view even if it differs from what others have said. Keep in mind that we are equally interested in negative and positive comments. At times negative comments are more helpful.

This session will be recorded because we do not want to miss any of your comments. People often say really helpful things in these discussions, and I cannot write fast enough to get them all down. We have had a brief introduction to remember each other’s names and roles but be assured of complete confidentiality. We will not use any names in our reports.

I would be happy to answer any questions you have after the group session. You can feel free to turn your camera on or leave it off, but please, can we raise a virtual hand if we want to say something or have a question? Let us kindly avoid talking over another person as much as possible, so we can hear everyone clearly.

P.S- Please if you haven’t signed your consent form, kindly do so and send it via email, thanks!

It is also important to mention that this focus group is solely for the purpose of service development and not for academic research, as the participant information may have insinuated.

Thanks, everyone, and let us begin….

**Schedule:**

***Engagement/Warm-up Questions***

1. What has your leadership training and mentorship experience as a Pharmacist been over your years of practice?
2. How would you describe your level of knowledge within AMS, and how was this knowledge attained?
3. Can you describe professional training opportunities (including CPD) in your country in one word?

***Exploration Questions***

1. What are your views about, and expectations for, the CwPAMS Leadership Programme?

(Probe questions- What would you say we need to focus on? If you were to pick one most important area for leadership development, what would it be?)

1. What are your thoughts about the selected areas for training: AMS, Health Leadership, Service Development, Pharmaceutical Public Health?

(Probe question- Do you think these reflect training needs in your country?

1. A pilot survey just conducted revealed some important areas within these domains **** would only read out a few (professional use of social media, risk management and change management, developing an antibiogram, diagnostic stewardship and surveillance and epidemiology of AMR, mental health, climate change, supply chain management, health systems strengthening as well as epidemic preparedness, involving nutrition support, population health and clinical research, programme evaluation, e-health, budgeting and cost-effectiveness as well as policy development, monitoring and evaluation (though this is already captured), social media and health communication, grant applications, setting up research projects, dose optimization, leadership models and styles) do these resonate with you as well?

(Probe question- Are there other areas you would wish for us to focus on?)

1. What do you think about us hosting this training on an online learning platform?

(Probe questions- Do you think this would be accessible? What benefits do you think this may have? What barriers do you foresee? What online learning methods do you recommend? What post-training assessment methods may be best?)

1. How do you think we can ensure that the CwPAMS Leadership programme is relevant to practice?

(Probe questions- Will the programme affect practice/ Is practice likely to change? Does its relevance matter? Is there anything that can be done to ensure this?)

1. What barriers and facilitators do you foresee to the success of this programme?

(Probe question- Do you anticipate barriers to effective project conduct at the end of the training?)

***Exit Questions***

1. Is there anything else you would like to say about what we have discussed so far? (Go around the table to ask everyone, and ensure everyone contributes)

(Probe question- Finally, of all the things we have discussed what is most important to you?)
